# Successful Early Diagnosis of Takotsubo Cardiomyopathy Induced by Status Epilepticus and Pneumonia Following the Exacerbation of Gastroesophageal Reflux Disease: A Case Report

**DOI:** 10.7759/cureus.71662

**Published:** 2024-10-16

**Authors:** Yasutoshi Yamamoto, Akihiro Matsushita, Sanae Watanabe, Katsuji Tanaka

**Affiliations:** 1 Pediatrics, Nishinomiya-Sunago Medical and Welfare Center, Nishinomiya, JPN

**Keywords:** gastroesophageal reflux disease (gerd), n-terminal pro-brain natriuretic peptide, pneumonia, status epilepticus (se), takotsubo cardiomyopathy (ttc)

## Abstract

Status epilepticus (SE)-induced Takotsubo cardiomyopathy (TTC) frequently manifests as a hemodynamic compromise, including shock, despite heart failure therapy. This can result in life-threatening events that pose significant diagnostic challenges. We report the case of a 57-year-old woman who was successfully diagnosed with TTC, without hemodynamic compromise, and treated.

Two years prior to TTC onset, the patient had exacerbated gastroesophageal reflux disease (GERD) due to recurrent hiatal hernia, accompanied by electrocardiogram (ECG) changes. On the day of TTC onset, the patient developed SE, as well as a high fever due to pneumonia. The SE was resolved by diazepam, and the patient received fluid therapy and antibiotics for the pneumonia. High creatine kinase (CK) and N-terminal pro-brain natriuretic peptide (NT-pro BNP) levels, along with ECG findings (ST elevation in left precordial leads), transthoracic echocardiogram findings (typical apical ballooning), and coronary computed tomography angiography findings (absence of culprit region), confirmed the TTC diagnosis. Although transient mild left ventricular outflow tract stenosis was observed on day 14, it disappeared on day 21. During the course of the disease, the patient received conservative management, with careful monitoring and follow-up imaging, and signs of hemodynamic compromise, such as shock, hypotension, or heart failure, were not observed.

The condition was triggered by SE and pneumonia following exacerbation of GERD. The extremely high NT-pro BNP level indicated that the CK elevation was due to myocardial damage rather than SE, facilitating early diagnosis. TTC should be considered when a patient presents with SE and pneumonia following GERD exacerbation and expresses remarkable NT-pro BNP elevation.

## Introduction

Takotsubo cardiomyopathy (TTC) is a reversible myocardial dysfunction that typically manifests as left ventricular apical ballooning without significant coronary artery disease. It is often triggered by emotional stress or various disorders, including neurological, gastrointestinal, and pulmonary diseases [1]. Although the detailed pathophysiological mechanism remains unknown, the stress induced by these conditions activates the sympathetic nervous system, causing excessive catecholamine release and subsequent myocardial damage, which leads to TTC. Status epilepticus (SE)-induced TTC frequently manifests as hemodynamic compromise and causes life-threatening events, making diagnosis challenging due to postictal consciousness disturbance and, rarely, chest pain [2,3].

Although cases of TTC triggered by SE, gastrointestinal disease, or pneumonia have been reported, no cases triggered by all these factors simultaneously have been documented [1,2,4-17]. Herein, we present a case of early diagnosis and treatment of TTC triggered by SE and pneumonia following gastroesophageal reflux disease (GERD) exacerbation, without hemodynamic compromise. Additionally, we highlight how N-terminal pro-brain natriuretic peptide (NT-pro BNP) measurement proved useful in early diagnosis.

## Case presentation

**Figure 1:**
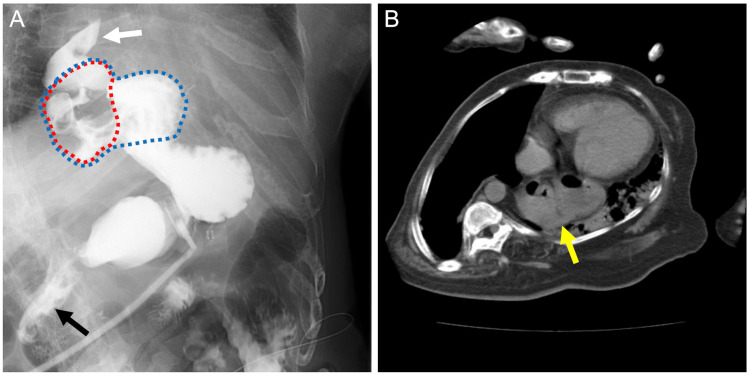
Gastrointestinal series and CT findings. (A) Upper gastrointestinal contrast imaging via a gastrostomy tube two years before this attack demonstrated the simultaneous appearance of the duodenum (black arrow) and esophagus (white arrow) due to a relapse of hiatal hernia (blue dotted circle), including prolapse of esophagofundal wrapping as a filling defect (red dotted circle). (B) CT scan at the onset of this attack indicated a hiatal hernia (yellow arrow). CT: computed tomography

**Figure 2:**
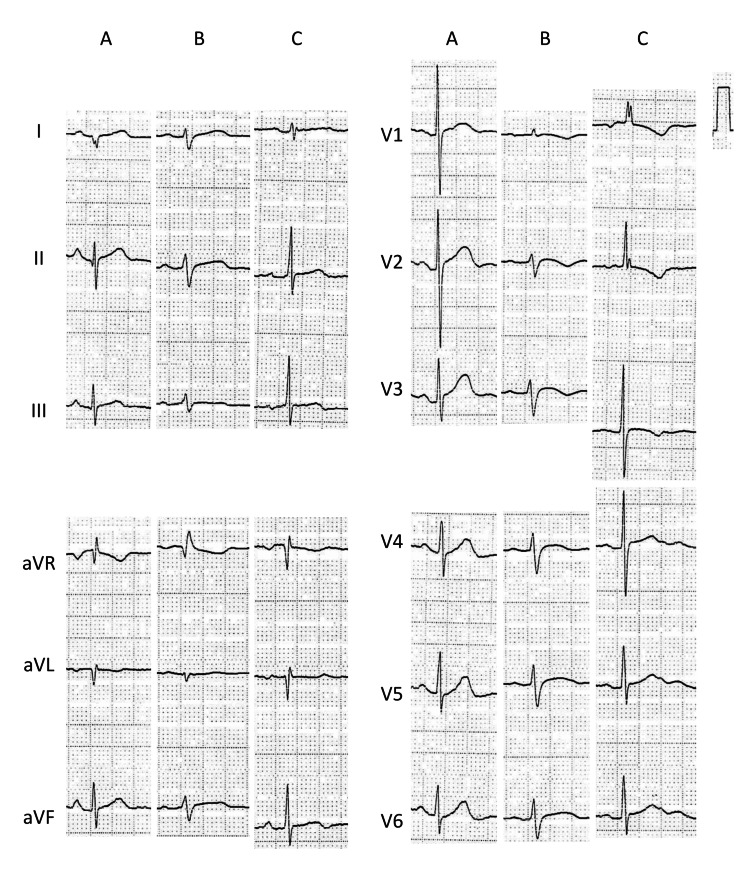
A 12-lead ECG findings at before onset, onset, and exacerbation of GERD. (A) ECG before the onset of GERD. (B) ECG at the onset of GERD demonstrated low QRS voltage. (C) ECG during the exacerbation of GERD demonstrated an inverted T wave at the precordial leads. ECG: electrocardiogram; GERD: gastroesophageal reflux disease

In May 2019, a 57-year-old woman with severe motor and intellectual disability associated with cerebral palsy due to fetal distress experienced a sudden onset of an epileptic seizure at our residential hospital. She had epilepsy from infancy and began taking antiepileptic drugs at the age of three years. Despite receiving 500 mg of carbamazepine daily for seizure control, she experienced seizures three to four times a year, with the most recent attack occurring two months before the present illness in March 2019. She also had a history of esophageal hiatal hernia repair (Nissen fundoplication) 19 years ago in October 2000, which recurred 10 years after surgery in April 2010. After she received an open gastrostomy in June 2003, postoperative peritonitis occurred. In July 2003, she underwent colectomy and enterostomy. The hernia worsened, leading to GERD, initially presenting with severe hematemesis two years ago in May 2017 (Figure 1A). A 12-lead electrocardiogram (ECG) showed low QRS voltage (Figure 2B). Despite treatment with a proton pump inhibitor, the patient experienced repeated gastrointestinal bleeding, including hematemesis, intragastric bleeding, and tarry stools, with episodes of remission followed by gradual exacerbation of GERD until the onset of TTC. The ECG findings showed an inverted T wave at the precordial leads (Figure 2C).

**Figure 3:**
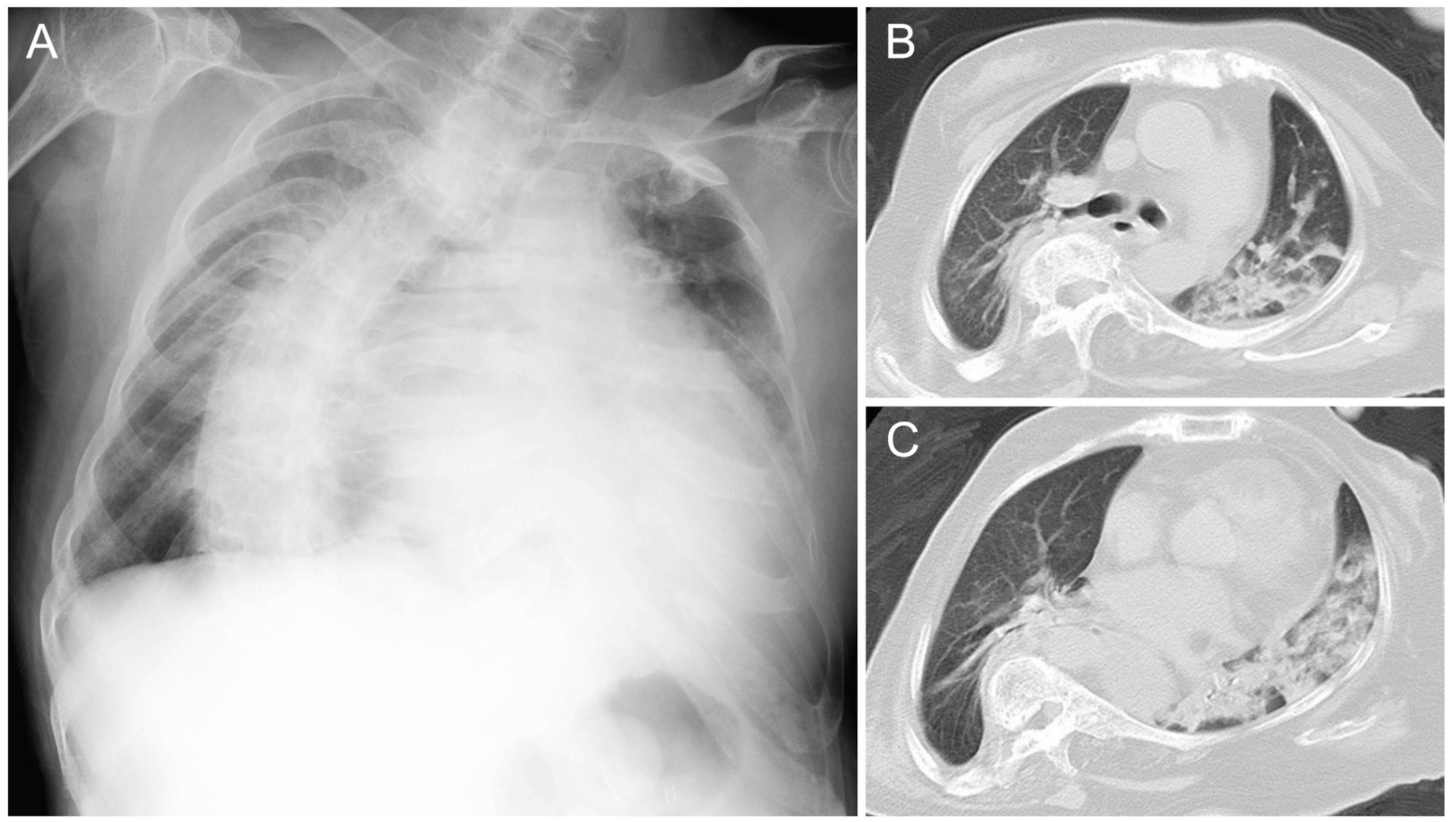
Chest X-ray and CT findings. (A) Chest X-ray showed cardiomegaly, left pulmonary infiltration, and scoliosis at the onset of illness. (B)-(C) CT demonstrated infiltration in both the left upper and lower pulmonary fields consistent with aspiration pneumonia at the onset of illness. CT: computed tomography

In May 2019, at 6:00 AM, she began to cry out strange sounds and had repeated short, generalized seizures with high fever. At 6:15 AM, she was diagnosed with SE because of unclear consciousness and incomplete seizure suspension. After suppository administration of diazepam, the seizures gradually halted. At 9:30 AM, laboratory data revealed inflammatory changes: white blood cell (WBC) count 8.2 x 10^9^/L (neutrophils 81.3%), hemoglobin level 13.3 g/dL, platelet count 263 × 10^9^/L, and C-reactive protein (CRP) level 2.5 mg/dL. At 10:00 AM, chest radiography showed left pulmonary infiltration indicating suspected pneumonia (Figure 3A). At 11:40 AM, computed tomography (CT) showed infiltration in both the left upper and lower pulmonary fields, consistent with aspiration pneumonia (Figures 3B-3C). Fluid therapy and antibiotic treatment with 4.5 g × 3 times of tazobactam/piperacillin hydrate (TAZ/PIPC) per day were initiated.

**Figure 4:**
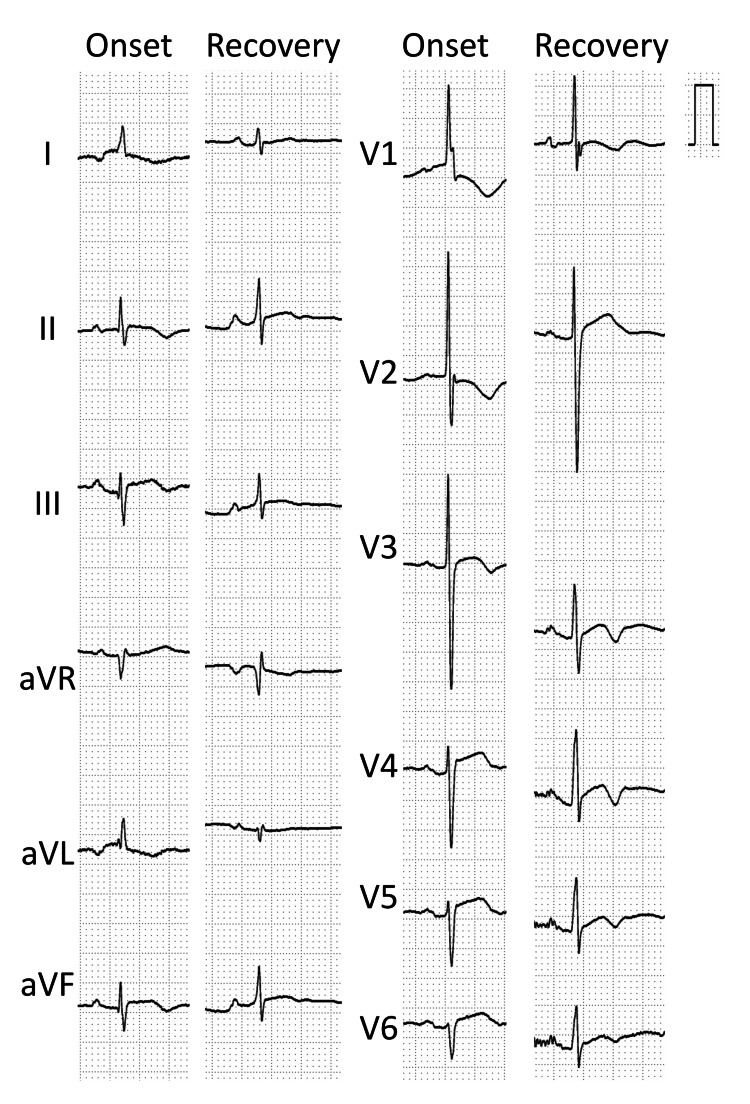
A 12-lead ECG findings at the onset of TTC and recovery period. ECG at the onset of illness showed normal sinus rhythm with ST-segment elevations in leads V4-V6 and negative T waves in leads I and II, with a positive T wave in aVR. TTC: Takotsubo cardiomyopathy; ECG: electrocardiogram; aVR: augmented Vector Right; aVL: augmented Vector Left; aVF: augmented Vector Foot

**Figure 5:**
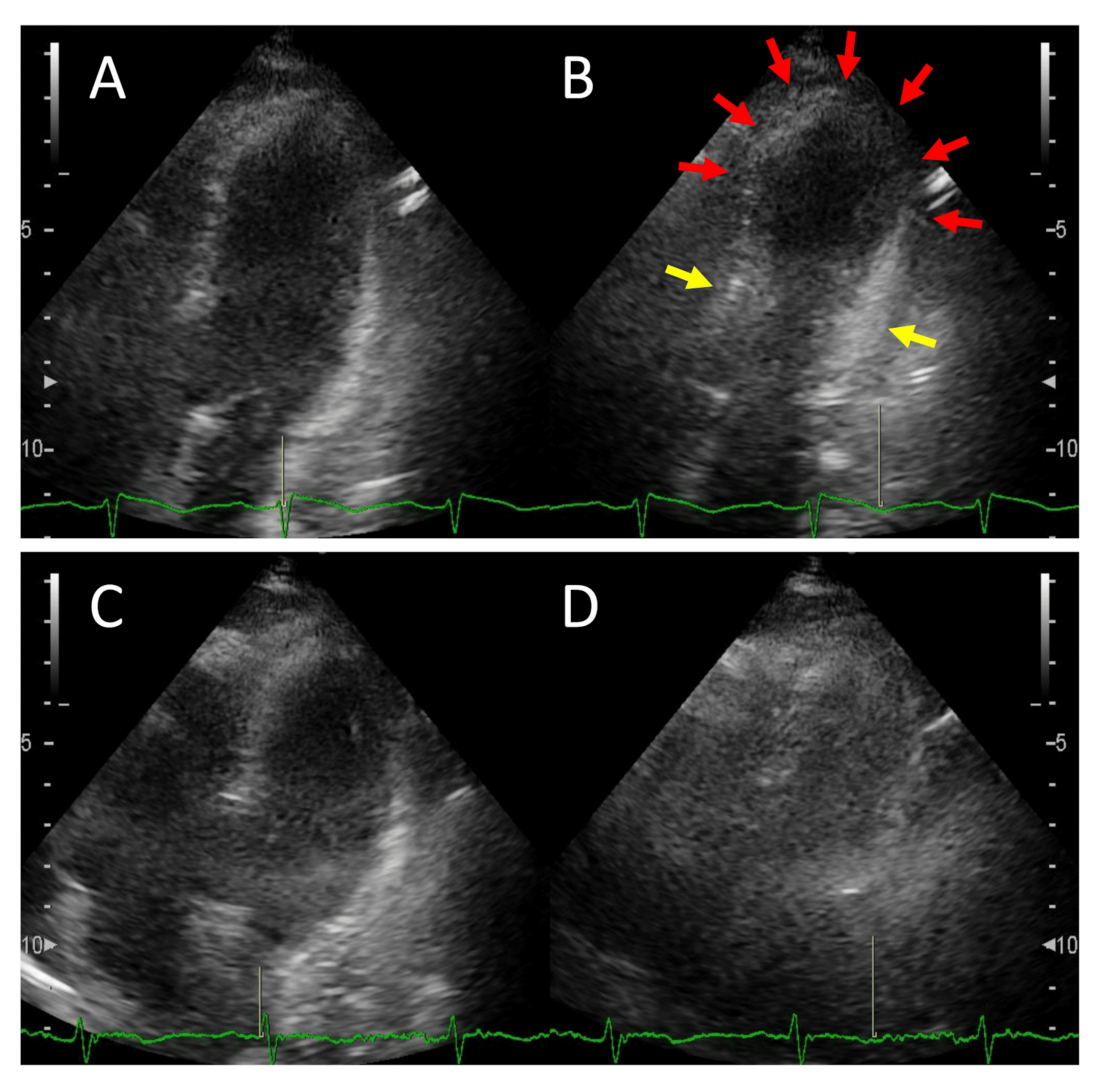
TTE findings at the onset of TTC and recovery period. Apical two-chamber view of TTE at the onset of TTC during diastole (A) and systole (B) showed left ventricular apical akinesia (red arrows) and preserved basal contraction (yellow arrows). TTE at the recovery period of TTC during diastole (C) and systole (D) demonstrated improved apical contraction. TTE: transthoracic echocardiogram; TTC: Takotsubo cardiomyopathy

At 1:00 PM, biochemical testing showed elevated creatine kinase (CK) at 1067 U/L (reference level: 30-172 U/L). At 1:30 PM, the ECG at the onset of illness demonstrated a normal sinus rhythm with ST-segment elevations in leads V4-V6 and negative T waves in leads I and II, along with a positive T wave in augmented Vector Right (aVR) (Figure 4). At 2:30 PM, transthoracic echocardiography (TTE) revealed left ventricular dysfunction with apical ballooning (Figures 5A-5B). After 5:00 PM, cardiac biomarkers showed remarkable elevations in the CK myocardial band (CK-MB) at 51 U/L (reference level: <25 U/L), cardiac troponin T (TNT) at 2.620 ng/mL (reference level: <0.014 ng/mL), and NT-pro BNP at 4334 pg/mL (reference level: <125 pg/mL), indicating that the CK elevation was not primarily due to SE but to myocardial damage. Coronary computed tomography angiography (CTA), performed on day 1 after the onset of illness, excluded coronary artery disease. The remarkable elevation of cardiac biomarkers, the ECG findings, typical TTE findings, and the absence of a culprit region on coronary CTA confirmed the diagnosis of TTC. Her cardiorespiratory status was stable, and she did not receive any cardiovascular agents, such as angiotensin-converting enzyme inhibitors, beta-blockers, or diuretics.

On day 2 after the onset of illness, the inflammatory response worsened: WBC count 16.5 x 10⁹/L (neutrophils 83.2%) and CRP 15.7 mg/dL. On day 7 after the onset of illness, sputum culture revealed group G beta streptococcus, which is susceptible to beta-lactam antibiotics. By day 8 after the onset of illness, the fever subsided and inflammation improved: WBC count 8.6 x 10^9^/L (neutrophils 72.0%) and CRP 1.87 mg/dL. We discontinued TAZ/PIPC on day 9 after the onset of illness.

**Figure 6:**
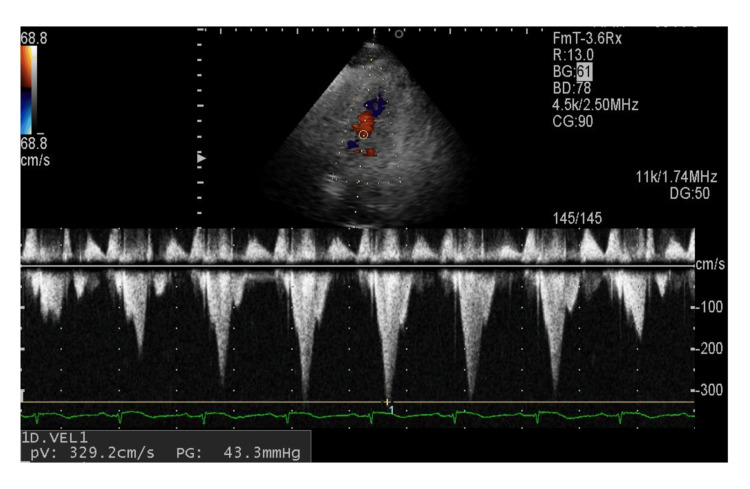
Doppler imaging in TTE findings. Continuous wave Doppler imaging in apical two-chamber view of TTE on day 14 showed a peaking dynamic pressure gradient of 43.3 mmHg in the left ventricular outflow tract. TTE: transthoracic echocardiogram

By day 12 after onset, both CK and cardiac TNT levels normalized, while NT-pro BNP levels were significantly elevated to 9005 pg/mL (from 4334 pg/mL at onset; reference level: <125 pg/mL). On day 14, mild left ventricular outflow tract obstruction (pressure gradient of 43.3 mmHg) appeared on TTE (Figure 6) and disappeared with normal left ventricular movement by day 21. During the course of the disease, the patient received conservative management with careful monitoring and follow-up imaging, and signs of hemodynamic compromise, such as shock, hypotension, or heart failure, were not observed. NT-pro BNP levels normalized by day 90, with no subsequent left ventricular dysfunction. By nine months after onset, the ECG findings returned to the pre-onset state.

## Discussion

We found two important clinical points: (1) TTC can be triggered by a combination of exacerbation of GERD, SE, and pneumonia; and (2) the measurement of NT-pro BNP levels is useful in the diagnosis of this condition.

First, TTC can be triggered by a combination of GERD exacerbation, SE, and subsequent aspiration pneumonia. Although approximately 1% of patients with SE develop TTC, the precise incidence and clinical background of this condition remain largely unknown [2]. Hemodynamic compromise is often observed in patients with SE-induced TTC, potentially leading to life-threatening events [3]. However, diagnosing SE-induced TTC is challenging due to frequent consciousness disturbances and the rare occurrence of chest pain [2,3]. Therefore, more cases may be overlooked, resulting in fatal outcomes [2,3]. Based on the literature review, we found 14 case reports and one article (including two recurrent cases [7,13]) related to SE-induced TTC [2,4-17]. The review of case reports and the article indicates the following: approximately 78% (14/18) of the patients with SE-induced TTC presented with hemodynamic compromise, including cardiogenic shock (nine), heart failure (three), hypotension (one), ventricular fibrillation (one), and atrial flutter (one). Only 5.6% (1/18) of patients with SE-induced TTC manifested chest pain, whereas all presented with consciousness disturbances due to SE. Approximately 61% (11/18) of the patients with SE-induced TTC had cardiovascular diseases as underlying health conditions, including hypertension (five), stroke (five), previous TTC (two), pacemaker implantation for sick sinus syndrome (one), and deep vein thrombosis (one). One case had pneumonia with patchy opacities [11], and only our case had both GERD and pneumonia (Table 1). To the best of our knowledge, this is the first reported case of TTC triggered by a combination of GERD exacerbation, SE, and subsequent aspiration pneumonia.

**Table 1:** Published cases of status epilepticus-induced TTC. “-” indicates absence; “+” indicates presence M: male; F: female; HF: heart failure; VF: ventricular fibrillation; HyT: hypotension; AF: atrial flutter; CVD: cardiovascular disease; PMI: pacemaker implantation; SSS: sick sinus syndrome; TTC: Takotsubo cardiomyopathy; HT: hypertension; DVT: deep vein thrombosis; GERD: gastroesophageal reflux disease; N/A: not available

Authors	Age/sex	Hemodynamic compromise	Chest pain	CVD	GERD	Pneumonia
Sakuragi et al. (2007) [4]	59/F	HF	-	PMI for SSS	-	-
Shimizu et al. (2008) [5]	75/F	Shock	-	N/A	-	-
Seow et al. (2008) [6]	62/M	Shock	-	-	-	N/A
Legriel et al. (2008) [7]	54/F	Two episodes (shock, shock)	-	Stroke, TTC	-	-
Fugate et al. (2009) [8]	82/F	Shock	-	HT	-	-
Traullé et al. (2011) [9]	50/M	HF	-	-	-	-
Finsterer et al. (2013) [10]	47/F	VF	-	-	-	-
Koo et al. (2015) [11]	83/F	Shock	-	Stroke	-	Patchy opacities
Uemura et al. (2016) [12]	61/F	-	-	HT, stroke	-	-
Srivastava et al. (2016) [13]	14/M	Two episodes (shock, shock)	-	TTC	-	-
Nandal et al. (2019) [14]	71/F	HyT	-	Stroke, DVT	-	-
Giovannini et al. (2019) [2]	74/F	-	+	HT	-	-
Giovannini et al. (2019) [2]	63/F	-	-	N/A	-	-
Al Fawaz et al. (2019) [15]	19/F	Shock, HF	-	-	-	-
Morinaga et al. (2020) [16]	82/M	-	-	HT	-	-
Hsiao et al. (2022) [17]	64/F	AF	-	HT, stroke	-	-
Our case	57/F	-	-	-	+	+

Second, the measurement of NT-pro BNP levels proved useful in diagnosing TTC triggered by a combination of GERD exacerbation, SE, and pneumonia. Previously, the usefulness of NT-pro BNP in diagnosing typical TTC was reported [18]. Both TNT and CK-MB are only slightly elevated in patients with TTC at onset, where they peak at day 1 and gradually decrease. In contrast, a remarkably high level of NT-pro BNP is often seen in patients with TTC at onset, which continues to increase after day 1. In our case, CK-MB was slightly elevated, and TNT was highly elevated at the time of onset, and they rapidly decreased after day 1. Likewise, NT-pro BNP was highly elevated at the time of onset and continued to increase after day 2, which was consistent with a previous report [18]. The NT-pro BNP level immediately increases after seizure by approximately 2.0 times compared to that of the control population (median: 130.0 pg/mL vs. control: 62.0 pg/mL) [19], whereas the level at the onset of TTC increases by approximately 27-fold (median: 1723 pg/mL) [18]. In our case, the NT-pro BNP level at the onset of TTC was abnormally high, increasing by over 60-fold (>4000 pg/mL), which facilitated early diagnosis. Thus, these findings suggest that the unique expression pattern of NT-pro BNP was also useful in the diagnosis of SE-induced TTC, a disease that could be easily overlooked.

In this case, we demonstrated that TTC could be triggered by multiple factors, including GERD exacerbation, SE, and pneumonia. Similarly, the occurrence of TTC triggered by multiple factors, rather than a single factor, has been proposed [20]. Izumi et al. reported that approximately half of the TTC cases with amyotrophic lateral sclerosis had precipitating factors, such as gastrostomy, tracheostomy, or infections [20]. They proposed the two-hit theory for the pathogenesis of TTC, where chronic cardiac sympathetic overactivity in amyotrophic lateral sclerosis acts as a predisposing factor, and acute infection serves as a precipitating factor [20]. Previously, GERD has been associated with linked angina, suggesting that non-cardiac disorders, such as esophageal dysfunction, can trigger myocardial-ischemia-linked angina through esophageal acid stimulation, resulting in reduced coronary blood flow [21]. Building on the two-hit theory and the concept of linked angina caused by GERD, we hypothesize a “multi-hit theory” for TTC pathogenesis. In this theory, chronic GERD-associated myocardial ischemia (referred to as linked angina) serves as the predisposing factor (first hit), SE-associated sympathetic overactivity acts as the precipitating factor (second hit), and aspiration pneumonia-associated sympathetic overactivity serves as an additional precipitating factor (third hit).

## Conclusions

TTC can be triggered by a combination of GERD exacerbation, SE, and pneumonia. Measurement of NT-pro BNP levels is useful in diagnosing this condition. We must consider TTC when a patient presents with SE and pneumonia following GERD exacerbation, and NT-pro BNP should be measured. Some cases of SE-induced TTC may be overlooked, suggesting the presence of more concealed cases. Therefore, NT-pro BNP should be more widely used for diagnosing TTC, especially after SE. Further research is needed to determine whether concealed SE-induced TTC is much more frequent and whether routine NT-pro BNP measurement could aid in the early diagnosis of TTC.
